# Citizen Science as an Approach for Responding to the Threat of *Anopheles stephensi* in Africa

**DOI:** 10.5334/cstp.616

**Published:** 2023

**Authors:** RYAN M. CARNEY, ALEX LONG, RUSSANNE D. LOW, SARAH ZOHDY, JOHN R. B. PALMER, PETER ELIAS, FREDERIC BARTUMEUS, LABAN NJOROGE, MAINA MUNIAFU, JOHNNY A. UELMEN, NIL RAHOLA, SRIRAM CHELLAPPAN

**Affiliations:** Department of Integrative Biology, University of South Florida (USF), Tampa, FL 33620, USA; Woodrow Wilson International Center for Scholars, Washington, D.C. 20007, USA; Institute for Global Environmental Strategies, Arlington, VA 22202, USA; US President’s Malaria Initiative, Entomology Branch, US Centers for Disease Control and Prevention, Atlanta, GA 30333, USA; Department of Political and Social Sciences, Universitat Pompeu Fabra, Barcelona 08005, Spain; Department of Geography, University of Lagos, Nigeria; Centre d’Estudis Avançats de Blanes (CEAB-CSIC), Blanes 17300, Spain; Centre de Recerca Ecològica i Aplicacions Forestals (CREAF), Cerdanyola del Vallès 08193, Spain; Institució Catalana de Recerca i Estudis Avançats (ICREA), Barcelona 08010, Spain; Section of Invertebrates Zoology, National Museums of Kenya, Museum Hill Road, Nairobi, Kenya; School of Pharmacy and Health Sciences, United States International University, Nairobi, Kenya; Department of Integrative Biology, University of South Florida (USF), Tampa, FL 33620, USA; MIVEGEC Unit, Montpellier University, IRD, CNRS, Montpellier, France; Department of Computer Science and Engineering, University of South Florida, Tampa, FL 33620, USA

**Keywords:** Africa, *Anopheles stephensi*, artificial intelligence, citizen science, malaria, mosquito

## Abstract

Even as novel technologies emerge and medicines advance, pathogen-transmitting mosquitoes pose a deadly and accelerating public health threat. Detecting and mitigating the spread of *Anopheles stephensi* in Africa is now critical to the fight against malaria, as this invasive mosquito poses urgent and unprecedented risks to the continent. Unlike typical African vectors of malaria, *An. stephensi* breeds in both natural and artificial water reservoirs, and flourishes in urban environments. With *An. stephensi* beginning to take hold in heavily populated settings, citizen science surveillance supported by novel artificial intelligence (AI) technologies may offer impactful opportunities to guide public health decisions and community-based interventions. Coalitions like the Global Mosquito Alert Consortium (GMAC) and our freely available digital products can be incorporated into enhanced surveillance of *An. stephensi* and other vector-borne public health threats. By connecting local citizen science networks with global databases that are findable, accessible, interoperable, and reusable (FAIR), we are leveraging a powerful suite of tools and infrastructure for the early detection of, and rapid response to, (re)emerging vectors and diseases.

## INTRODUCTION

Malaria is a disease caused by *Plasmodium* parasites that are transmitted by a subset of female mosquitoes in the genus *Anopheles*. This disease was first documented as a health threat as early as 3200 BCE and has been plaguing humanity ever since (Institute of Medicine [US] 2004). In 2020, malaria cases worldwide rose to 241,000,000 – killing 627,000 people, almost half a million of whom were children under the age of five (World Health Organization [WHO] 2021). While there are five species of the parasite that can lead to human malaria, *Plasmodium (P.) falciparum* and *P. vivax* are the two of primary concern in Africa—the continent with 95% of cases and 96% of deaths (WHO 2022a). There are 30–40 species of *Anopheles* mosquitoes known to transmit human malaria, but the principal vectors in Africa are the *Anopheles (An.) gambiae* complex and *An. funestus* group (Centers for Disease Control and Prevention [CDC] 2020). Given that these indigenous vectors thrive in natural habitats of standing water, such as puddles and rice paddies, malaria has previously been a predominantly rural disease (Sinka et al. 2020; Villena et al. 2022).

Although malaria is already a substantial burden in Africa, the situation has now become even more complicated and urgent with the recent discovery of an invasive species, *An. stephensi*, present throughout the Horn of Africa and beyond (WHO 2022b). What is especially troubling about these findings is the urban nature of *An. stephensi*, as this mosquito has the ability to reproduce and thrive in artificial water storage containers (Surendran et al. 2019), which are plentiful in areas without piped water. A characteristic of urban development in Africa has been its unplanned and unstructured nature, where residential buildings are crammed into small areas without accompanying infrastructure such as pathways, drainage, or waste disposal points. These factors result in a myriad of potential breeding locations for *An. stephensi* following rainfall episodes. Further exacerbating this threat, *An. stephensi* has been found to be a highly competent vector of both *P. falciparum* and *P. vivax* (Tadesse et al. 2021) and highly resistant to nearly all the insecticides used in malaria mosquito control (Balkew et al. 2021). Considering this species’ great capacity for proliferation and disease transmission, understanding its distribution throughout the continent—and especially in new areas—is of utmost importance.

## THE *ANOPHELES STEPHENSI* PROBLEM

This species was previously thought to be confined to urban areas in southern Asia and the Arabian Peninsula and absent in the Horn of Africa. However, in 2012, *An. stephensi* was discovered in association with an outbreak of malaria in Djibouti City (Djibouti), which resulted in urgent calls for increased surveillance and tracking of this invasive vector (Faulde et al. 2014). Just three or four years following its detection in Djibouti and subsequently in Ethiopia, populations of *An. stephensi* were found to persist year-round, unlike indigenous vectors, creating opportunities for sustained and significant malaria outbreaks at unprecedented times of the year (de Santi et al. 2021, Tadesse et al. 2023). Such a scenario seems to have played out in Djibouti, where reported cases of malaria rose from only 27 to 73,535 between 2012 and 2020 (WHO 2021). Similarly, recent data from Ethiopia demonstrate that *An. stephensi* drove transmission of a malaria outbreak during the dry season in Dire Dawa in 2022 (Tadesse et al. 2023).

By 2023, invasive *An. stephensi* had been reported in Djibouti, Eritrea, Ethiopia, Ghana, Kenya, Nigeria, Somalia, and Sudan, as well as Sri Lanka and Yemen (WHO 2023a; Ochomo et al. 2023). This range expansion means that more and more new communities require immediate public health interventions. Specifically, mitigating the spread of this vector relies upon early detection and sustained surveillance throughout the year (Whittaker et al. 2023), coupled with rapid response prior to full establishment, whereupon elimination becomes difficult if not impossible. There is also the danger of this vector reestablishing malaria in regions where the disease had previously been eradicated. If left unchecked, the spread of *An. stephensi* on the African continent alone is estimated to put an additional 126 million people at risk of malaria (Sinka et al. 2020).

This accelerating risk is made clear by the September 2022 announcement of the *WHO initiative to stop the spread of* Anopheles stephensi *in Africa* (WHO 2022c), following the WHO’s first vector alert concerning *An. stephensi* in 2019 (Global Malaria Program 2019). The announcement was made as pressure mounted to address the new threats to urban areas that *An. stephensi* poses. This initiative takes a five-pronged approach:

1. “increasing collaboration across sectors and border[s];
2. strengthening surveillance to determine the extent of the spread of *An. stephensi* and its role in transmission;
3. improving information exchange on the presence of *An. stephensi* and on efforts to control it;
4. developing guidance for national malaria control programmes on appropriate ways to respond to *An. stephensi*;
5. prioritizing research to evaluate the impact of interventions and tools against *An. stephensi*” (WHO 2022c).

The WHO initiative also recommends that national responses be guided by four pillars laid out in its global vector response framework, including “engaging and mobilizing communities” and “scaling up and integrating tools and approaches” (WHO 2017). These priorities build on the no-longer-novel idea that to effectively monitor mosquitoes and manage outbreaks, community-centered innovations must be considered.

**Figure 1:**
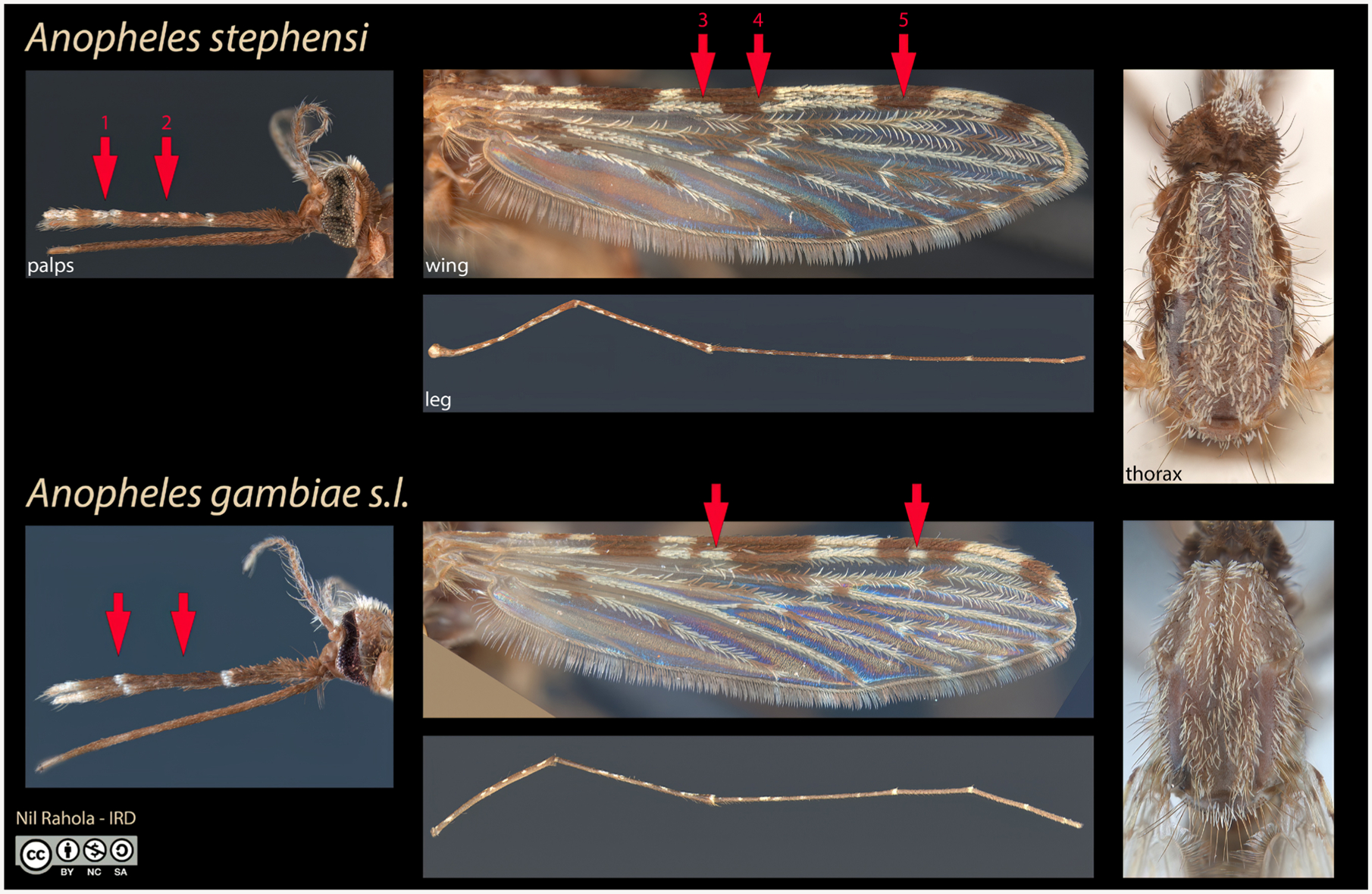
Diagnostic features of adult female *Anopheles* (*An*.) *stephensi* compared to *An. gambiae* sensu lato. On the palps, *An. stephensi* has a broader subapical white band (red arrow #1) and white speckling (#2). On wing vein 1, the median dark spot has two pale interruptions (#3,4), sometimes fused with the presector pale spot to the left and the subcostal pale spot to the right, respectively (as they are here). The subapical dark spot has no pale interruption (#5). Additionally, the scales of the thorax are broader in *An. stephensi* (*top right*). Legs are practically indistinguishable.

With respect to monitoring *An. stephensi* in particular, one key problem is the fact that identification of this species in the adult stage (Figure 1) and especially the larval stage is challenging. Additionally, adult-collection methods such as CDC light traps and pyrethrum spray catches are unreliable and insufficient for *An. stephensi* (US President’s Malaria Initiative [PMI] 2023). Even if efficacy were not an issue, traditional mosquito-collection methods are expensive, require expertise and time, and are difficult to implement across large areas and jurisdictional boundaries. However, citizen science surveillance can be cost-effective and scalable, and can provide comparable quality and predictive power (Palmer et al. 2017).

## THE CITIZEN SCIENCE SOLUTION

The aforementioned goals can be better realized by incorporating projects that harness citizen science, also known as community science, via mobile app-based tools and campaigns that augment, motivate, and support community-centered mosquito surveillance programs. Members of the public can contribute useful data by taking photographs of mosquitoes, locating and mitigating oviposition (breeding) sites, and documenting the presence of larvae, pupae, or mosquito bites. Such real-time information can allow new vector populations to be more nimbly detected as they arise, and apps such as these can be used in any community where there is a smartphone accessible. Citizen science thus has the potential to leverage the efforts of the general public to obtain the volume, velocity, and variety of data needed at local, continental, and global scales, in order to inform mosquito control strategies and to improve our predictive models of vector-borne disease. A recent literature review has identified citizen science projects that provide effective mosquito surveillance and operate on various spatial scales, and many of these have reported actionable outcomes that support mosquito control strategies (Sousa et al. 2022). The three international mobile app projects comprising the backbone of our coalition’s efforts are described below.

### GLOBE OBSERVER

A promising approach to expanding the reach of citizen science-based mosquito surveillance is collaborating with partners such as GLOBE (est. 1995), an international organization created through bilateral country agreements with the US Department of State. Specifically, NASA’s GLOBE Observer mobile app (observer.globe.gov) is available for use in 127 countries and has been translated into 15 languages. The GLOBE program has a long track record of engaging students and citizen scientists, with more than 200 million observations logged since 1995 (Amos et al. 2020; Low et al. 2022). User-provided data includes information on and photos of mosquito larvae, pupae, and breeding habitats collected using the Mosquito Habitat Mapper tool. Through the Land Cover tool, ecological information and photos are collected, which can complement the mosquito data in subsequent analyses; participants are prompted through the app to take such coincident observations after using the Mosquito Habitat Mapper tool.

Historically, creation of the Mosquito Habitat Mapper tool was stimulated by the GLOBE community in Africa, who developed the initial protocol for local use in the 1990s. This work was then incorporated into the GLOBE Program as a scientifically vetted international protocol in 2014. The digital app was codeveloped with GLOBE partners in Thailand and Africa in 2016, and was first tested in partnership with communities in Peru and Brazil in 2017, in response to the previous year’s epidemic of Zika. The app particularly targeted the vectors of that disease, *Aedes* (*Ae*.) *aegypti* and *Ae. albopictus*. In Africa, GLOBE citizen science has provided actionable data for mosquito control, for example by demonstrating that the most frequent oviposition sites vary among three communities in Senegal (Low et al. 2021). In Latin America, access to and use of citizen science data collection apps have proved to be motivating, especially for adolescents, and the Cooperative for Assistance and Relief Everywhere’s “Juntos ante el Zika” (Together Fighting Against Zika) project incorporated GLOBE Observer Mosquito Habitat Mapper training and use into youth-led community mosquito-awareness programs. Internationally, behavioral data collected using the GLOBE Observer Mosquito Habitat Mapper app has shown that the majority of citizen scientists who collected data were also motivated to reduce the availability of breeding sites by removing, draining, or covering water containers where possible (Low et al. 2021).

### MOSQUITO ALERT

**Figure 2:**
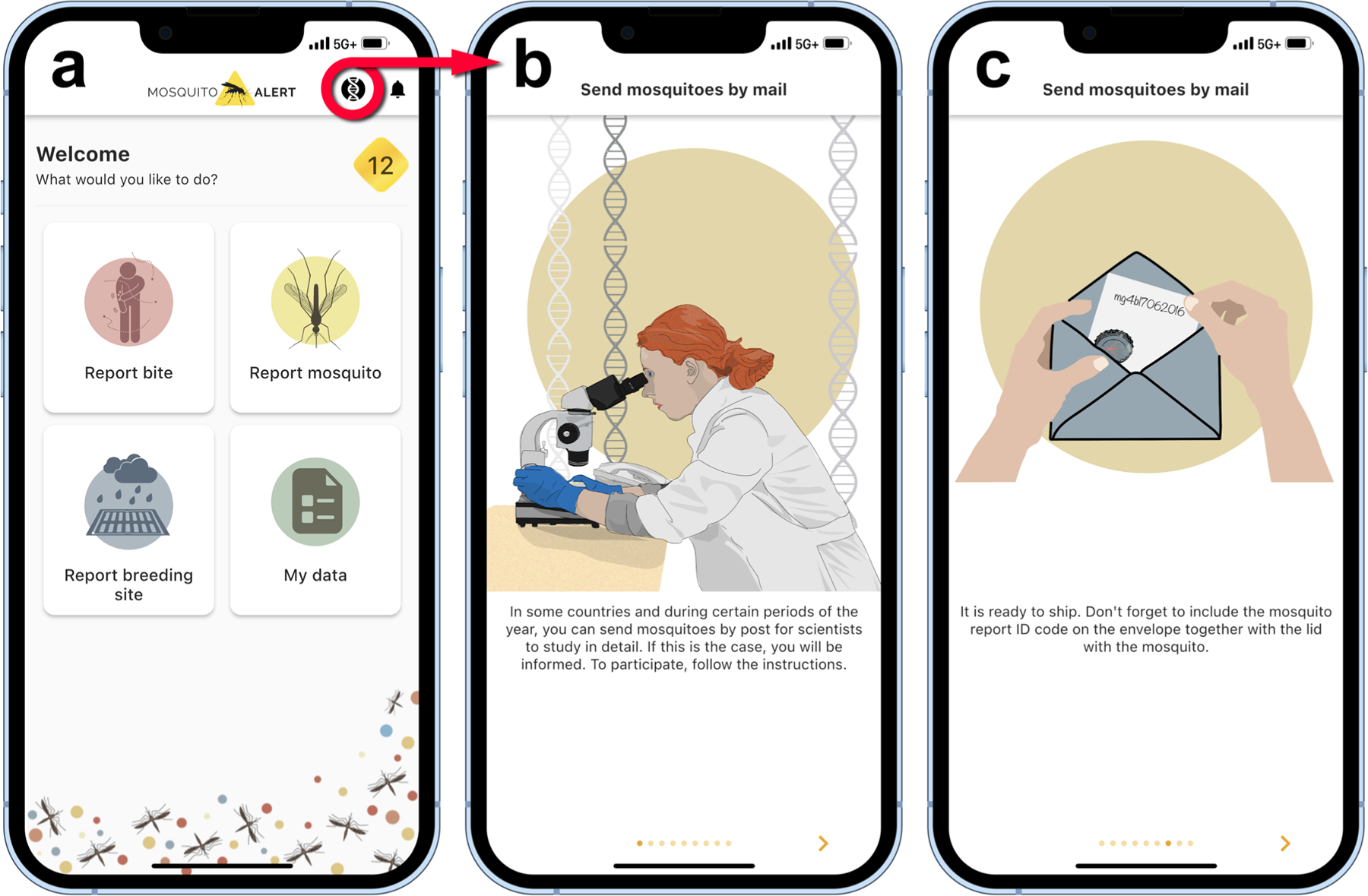
Mosquito Alert user interface. Screenshots illustrating the mobile app’s dashboard **(a)**, where clicking on the DNA icon (*red circle*) will provide instructions **(b)** for mailing a mosquito specimen for testing **(c)**, provided the citizen scientist is in a country with a partner laboratory actively accepting submissions.

Mosquito Alert (mosquitoalert.com) is an international citizen science project involving the submission of photos of adult mosquitoes as well as information on mosquito bites and breeding habitats (Figure 2a). The app has been translated into 19 languages, including languages broadly spoken in Africa, such as French and English. The app provides instructions on taking the mosquito photos so they are suitable for species identification and subsequent expert validation (e.g., “Try to photograph the mosquito so that the markings on its thorax and legs can be seen;” https://www.mosquitoalert.com/en/project/envia-datos/tips-for-your-photos/). As a form of gamification to incentivize participation, citizen scientists are awarded points based on the type of mosquito and breeding site reported, as well as extra bonuses for actions such as participating frequently. The accumulation of points allows users to reach five different levels and be placed on a leaderboard with global rankings of users. There are also trophies based on various milestones. In addition, Mosquito Alert has created customized management portals and alerts, for communicating directly with agencies and users (Bartumeus et al. 2018), as well as data-visualization tools such as vector risk maps (e.g., https://labs.mosquitoalert.com/MosquitoAlertES/).

Since its inception in 2014, Mosquito Alert has demonstrated how citizen scientist action has improved understanding of the movement and location of vector species in Spain, compared with that of municipal mosquito control programs alone (Palmer et al. 2017). It is often the case that participants do not send pictures of targeted species but of similar ones or of species that are strange to them. Indeed, the discovery of *Ae. japonicus* in the north of Spain in 2018 has demonstrated that citizen scientists can report species of public health interest other than the species identified *a priori* by scientists (Eritja et al. 2019; Eritja et al. 2021). *Ae. japonicus* was not included in the initial versions of Mosquito Alert deployed in the country because, contrary to the information on *Ae. aegypti*, there were no previous records of this species in Spain. *Ae. japonicus* appeared for the first time in 2000 in eastern France, and dispersed to Belgium, Switzerland, and Germany (2008), and from there to other northern and eastern European countries (Austria, Slovenia, Netherlands, Hungary, Croatia), to finally appear in Italy and Liechtenstein in 2015. The 2018 discovery reported by a Mosquito Alert participant in the north of Spain was validated in the field by the Spanish Ministry of Health, enhanced further citizen-based monitoring, and activated different field surveillance strategies by experts in the area (Eritja et al. 2021). In addition, the species was added as one of the target species in the citizen science-based system.

There are plans for Mosquito Alert to redesign its data collection system to include, among other new features, the genus *Anopheles*, so as to better fight malaria in Africa. Even if *An. stephensi* is not explicitly included in the app’s mosquito guide, initiating deployments of the app in Africa would be useful.

### iNATURALIST

The social networking platform iNaturalist (inaturalist. org) is available via mobile app and web browser and in more than 50 languages, including Arabic, French, and Portuguese. Users submit photos (or sound recordings) of wild organisms, along with a date and location data. These organisms are then identified by other users, and at least two-thirds of these crowdsourced taxonomic classifications must agree for the record to be designated as “Research Grade.” (To date, only one Research Grade observation of *An. stephensi* exists on the iNaturalist platform, which was recorded in Kuwait in November 2021). Users can also subscribe to taxa, locations, and other users, which provides daily updates on submissions of interest and thus a means of monitoring natural populations.

**Figure 3:**
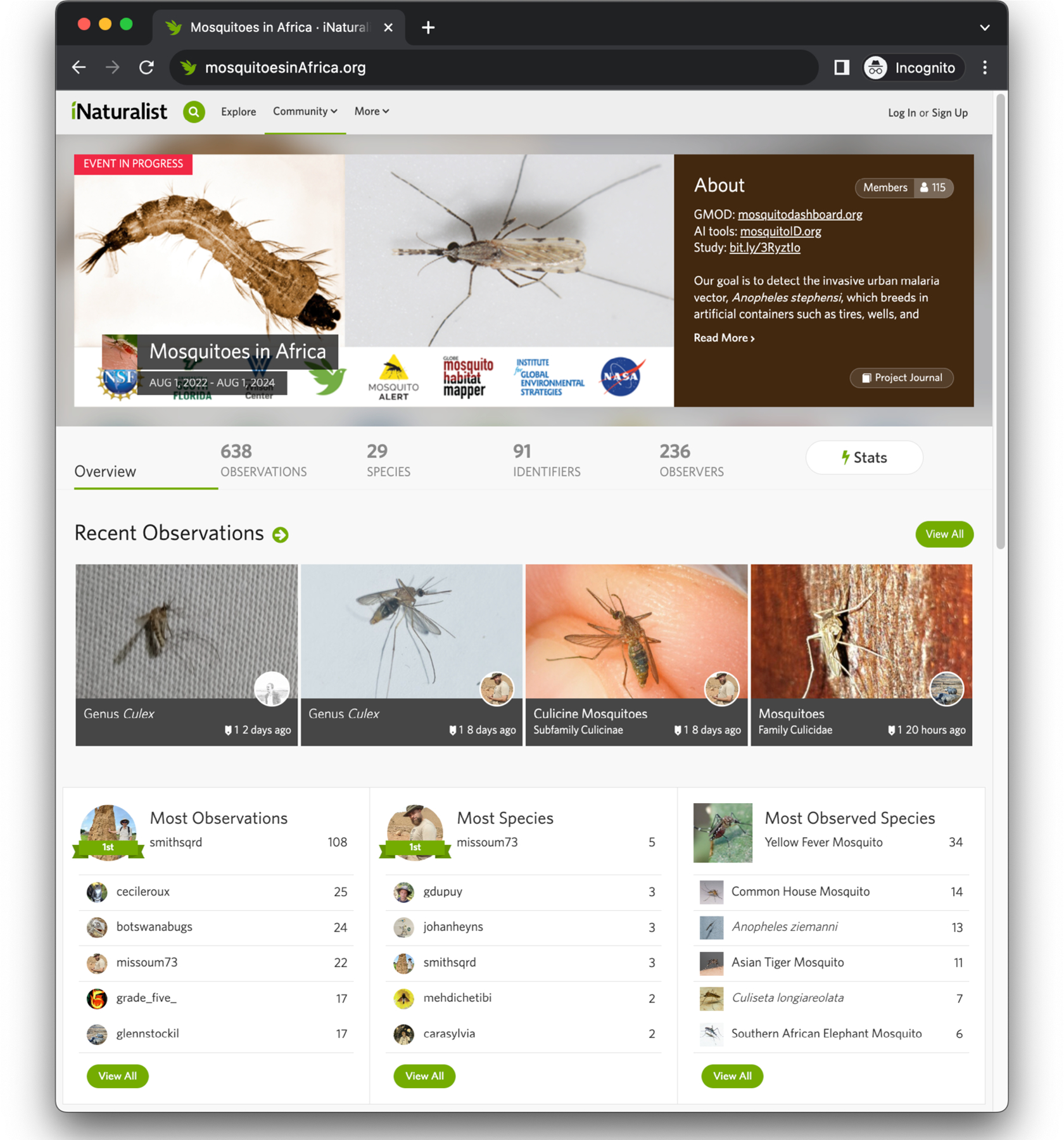
Our ongoing iNaturalist citizen science campaign for monitoring mosquitoes throughout Africa, particularly targeting *Anopheles stephensi* (mosquitoesinAfrica.org).

Further platform engagement and promotion can occur through coordinated “bioblitzes,” which are community events targeting one or more species at a given location for a short period, or through project pages that cover larger geographic areas and/or longer spans of time, and include leaderboards for the number of observations and identifications (Figure 3). Our previous efforts on this platform have included various targeted citizen science campaigns in the Americas (mosquitoAI.org), which yielded detections of invasive vector mosquitoes in new areas (Carney et al. 2022) and validation data for a mosquito habitat model (Uelmen et al. 2023). More recently, we have launched a campaign in Africa focused on *An. stephensi* (mosquitoesinAfrica.org; Figure 3). These observations have already led to the discovery of a previously undocumented phenomenon, mosquito bite-induced color change in chameleon skin (Garcia et al., in revision), demonstrating the utility and serendipity of citizen science observations.

### THE GLOBAL MOSQUITO ALERT CONSORTIUM

These successes observed in local and regional citizen science surveillance projects suggest the importance of collaboration in the scientific community to make data, tools, and expertise available worldwide. This is especially true for low-resource communities that can benefit from the use of ready-made, widely available data collection apps, a cloud-based archive, and open-source analysis tools developed for the greater good by others and available at no cost.

This was the vision behind the creation of GMAC. Hosted by the Woodrow Wilson International Center for Scholars (Wilson Center), the European Citizen Science Association (ECSA), and the United Nations Environment Programme, representatives from seven citizen science programs focused on mosquito surveillance convened in 2017 to envision an inclusive international effort to support mosquito surveillance and mitigation (Tyson et al. 2018; Palmer et al. 2018). The expressed goal of this project was to provide a digital infrastructure that could harness rapidly emerging open-data tools and capabilities, together with mosquito data from across the globe. Subsequent to this meeting, significant steps have been made to make this vision a reality.

**Figure 4:**
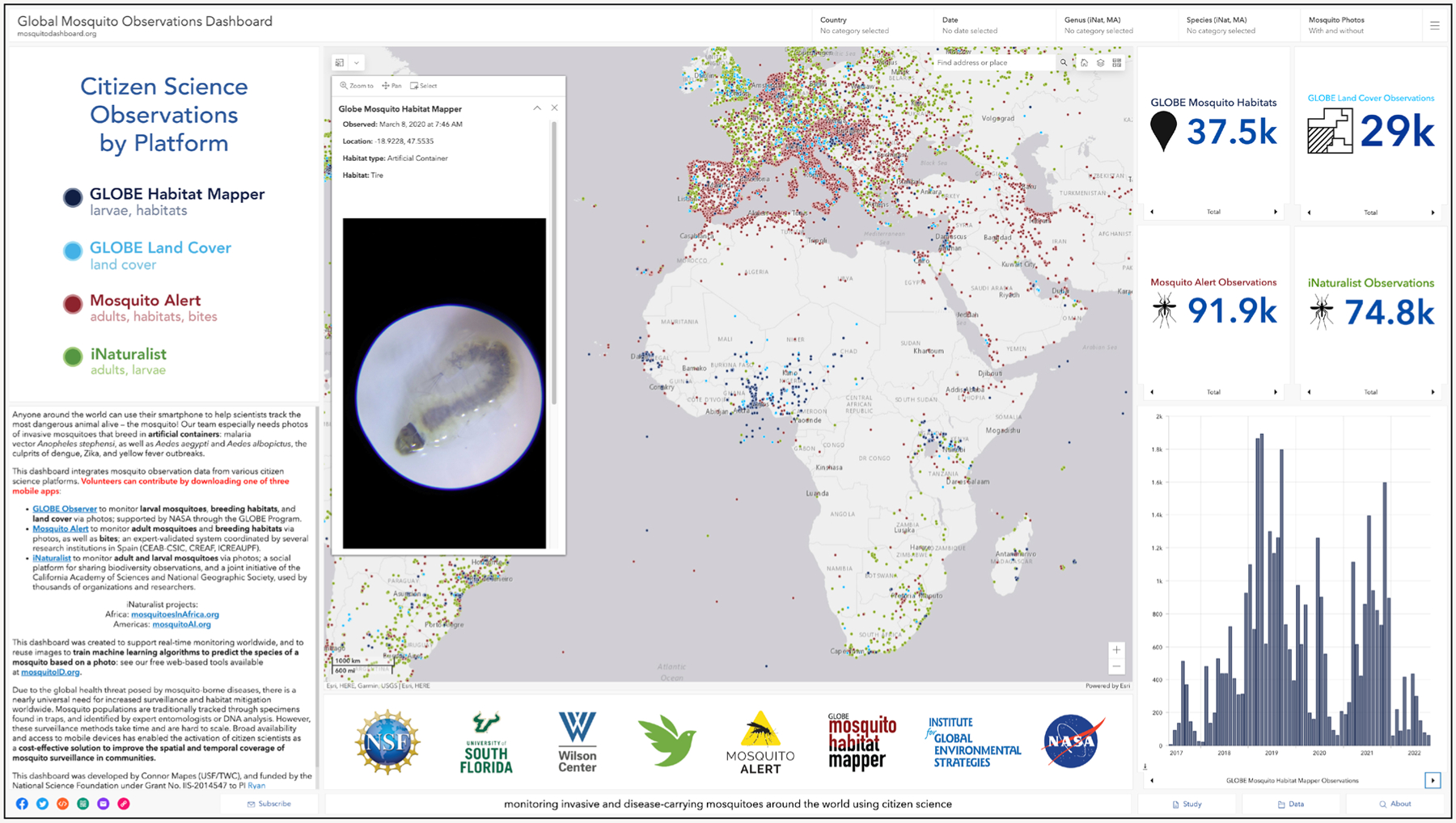
Global Mosquito Observations Dashboard, desktop interface (mosquitodashboard.org); which integrates datasets from four global platforms: Mosquito Alert, iNaturalist, and NASA GLOBE Observer’s Mosquito Habitat Mapper (MHM) and Land Cover. Inset: MHM citizen scientist’s photo, taken with a smartphone and a 60× clip-on lens, of an anopheline larva found in a tire in Madagascar in March of 2020, classified by artificial intelligence (AI) algorithms as *An. stephensi*.

Through a GMAC collaboration, multiple international citizen science platforms—Mosquito Alert, Mosquito Habitat Mapper, Land Cover, and iNaturalist—were aligned to the Open Geospatial Consortium data interoperability standards (Ingle et al. 2021) and harmonized into the Global Mosquito Observations Dashboard (GMOD; mosquitodashboard.org; Figure 4; Carney et al. 2022). With this open and findable, accessible, interoperable, and reusable (FAIR) infrastructure now in place, it is possible to promote worldwide participation in mosquito surveillance to better meet scientific and public health goals. The large and increasing number of observations displayed on the GMOD (~300 K at time of print) highlights the already-established international usage of these apps for tracking mosquito populations.

This work was conducted across multiple research organizations, private collaborators, and government entities, including the University of South Florida, the Wilson Center, the US Department of State, the Institute for Global Environmental Strategies, Universitat Pompeu Fabra, Spanish National Research Council, NASA, iNaturalist, and many more. The sheer breadth of collaborators involved in this work should indicate how the integration of even relatively similar citizen science projects takes ample resources, international partnerships, and time. GMAC and its products are realizing the vision of empowering decentralized, local citizen science communities with open tools. These tools enable a broad cross-section of the world’s citizens to contribute to mosquito monitoring and public health in their own communities and to play a role in the global fight against diseases transmitted by mosquitoes.

## NEXT-GENERATION SURVEILLANCE OF *ANOPHELES STEPHENSI*

As Africa is undergoing multiple *An. stephensi*–driven malaria outbreaks, innovative forms of surveillance are necessary. The free products (apps, dashboard, protocols) and global connections fostered by the GMAC may offer such tools and an additional network to leverage for tracking the spread of *An. stephensi*.

With respect to materials for monitoring *An. stephensi* in particular, one of the key challenges is that this species can be easily confused with similar-looking congenerics. Therefore, as a complement to a recent key that includes this taxon (Coetzee 2020), we provide an identification reference image to assist citizen scientists and scientists alike in distinguishing between adult females of *An. stephensi* and the *An. gambiae* complex (Figure 1). The diagnostic markings on the mouthparts and wings are observable via a smartphone with a 60× clip-on lens. However, smartphone photos of adult mosquitoes—unlike the larvae—need not be taken through a 60× clip-on lens for submission to the aforementioned apps or for the artificial intelligence (AI) analysis below.

Another current challenge is that validation of citizen scientist mosquito identifications is critical for mosquito data to be scientifically useful. The Mosquito Alert platform employs expert communities to validate and annotate mosquito photos submitted by citizen scientists (Južnič-Zonta et al. 2022) and is increasingly turning to AI as a scalable strategy (Pataki et al. 2021; Carney et al. 2022). Volunteer entomologists validate the species on a highly heterogeneous timeframe of days to months, depending on their availability to log onto the platform (which may be poor when they are busy with their regional surveillance). We plan to reduce this bottleneck by means of an AI that can analyze and annotate a percentage of photos with an identification score, assigning them as “not classified” if below the threshold of confidence. Although this process may produce some false positives, it will mean that we can immediately return information to citizen scientists and add observations to the public map. It will thus decouple the process from the slower but more accurate validation procedure followed by human experts, who will still revise the results as needed later on.

**Figure 5:**
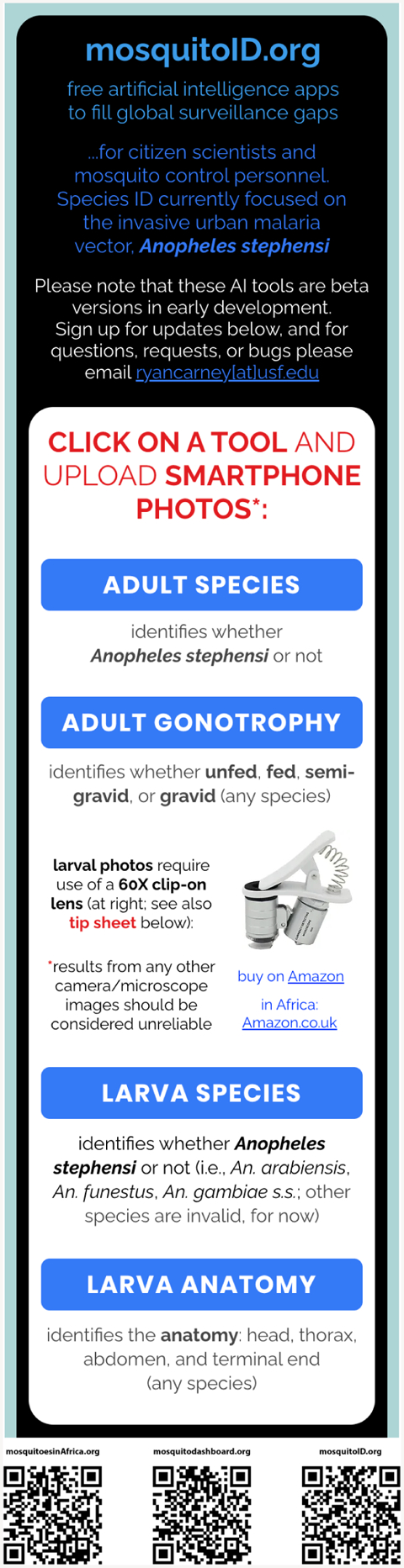
Artificial intelligence dashboard, mobile interface (mosquitoID.org). Note inset with 60× clip-on lens at middle right. Bottom: QR codes to direct readers to the respective websites.

A related opportunity is our development and deployment of novel AI methods to identify *An. stephensi* in particular. Specifically, these algorithms identify the genus and/or species of larval or adult mosquitoes using smartphone photos submitted directly by citizen scientists (Minakshi et al. 2020a, 2020b; Carney et al. 2022, in preparation). Other algorithms identify the larval anatomy and adult gonotrophic stage (unfed, fed, semi-gravid, gravid) (Azam et al., in revision). Results include image heat maps for explainability and validation. Free beta versions of these AI algorithms are available online (mosquitoID.org; Figure 5) for others to use in surveillance. The algorithms are also used by our team to automatically process the incoming citizen science images of larval and adult mosquitoes from Mosquito Habitat Mapper, Mosquito Alert, and iNaturalist on a daily basis, to detect potential *An. stephensi*. These AI efforts are ongoing, and more work is being done to expand the training-image database and to test the model’s accuracy through molecular testing and further validation exercises. In its final form, this method could complement the need for DNA sequencing and allow for more rapid alerting of local authorities to *An. stephensi* presence and abundance.

Larval surveillance—especially around human dwellings and animal shelters/pens—is especially important for *An. stephensi* (Ahmed et al. 2022; US PMI 2023), and such efforts should utilize the Mosquito Habitat Mapper tool. In Africa, this process has already been piloted through our citizen science activities in countries such as Ethiopia and Madagascar. In Madagascar, a photo of an anopheline larva from a tire—in conjunction with a total of > 100 anopheline larvae reported from three other artificial containers—was submitted to the app in March 2020 (Figure 4, inset). Subsequent classification as *An. stephensi* by our AI (Carney et al., in preparation) prompted immediate, sustained, and targeted local surveillance at and around the original site, starting in 2022, along with training workshops and the distribution of promotional flyers in English and French, as well as of 60× clip-on lenses. Since that year, similar citizen science activities have also been integrated into the formal surveillance efforts to detect *An. stephensi* in nearby Mauritius (Iyaloo et al. 2022).

## THE STATE OF CITIZEN SCIENCE IN AFRICA

For scaling the deployment of such efforts throughout Africa, it is important to acknowledge lessons learned from previous related efforts, as well as the current landscape of citizen science in general. Given the need for improved mosquito monitoring to successfully control malaria (Mnzava et al. 2014), researchers in Rwanda have proposed and subsequently investigated citizen science approaches for complementing existing vector surveillance and health networks (Murindahabi et al. 2018, 2021; see also companion paper on human behavior, Asingizwe et al. 2018). Results demonstrated that citizen science efforts can provide valuable information on the bionomics of the vector as well as on the spatial and temporal variation in the risk of malaria, all of which can contribute to the planning and cost-effective deployment of mosquito control strategies (Murindahabi et al. 2021). The authors also emphasized the central role of community health workers, especially in providing the critical link between government stakeholders and communities (Murindahabi et al. 2018). Similarly, community knowledge and experiences were harnessed to accurately assess the density and distribution of mosquito populations in three rural villages in Tanzania (Mwangungulu et al. 2016).

These efforts exemplify what Ashepet et al. 2021 identified as the three contributions that citizen science can make toward vector-borne diseases in Africa: boosting data collection, tapping into local knowledge, and building durable partnerships between communities and scientists. In turn, such contributions can help to overcome the three major challenges of controlling vector-borne diseases on the continent: insufficient mosquito data and experts, absence of sociocultural perspectives, and reactive short-term approaches implemented from the top-down (Ashepet et al. 2021). More generally, these authors also found that few African citizen science studies are focused on vector-borne diseases or public health, with the preponderance of studies focused on biodiversity/ecology (as well as clustered in East and South Africa) (Ashepet et al. 2021).

Citizen science on the continent also lacks a comprehensive database to show the existence, activities, and relevance for various United Nations sustainable development goals (SDGs). Therefore, the International Science Council’s Committee on Data Task Group on the SDGs undertook a study in 2020 to understand the landscape of citizen science projects in Africa and their potential contributions to the SDGs, with a focus on clean water and sanitation (SDG 6) and sustainable cities (SDG 11)—both topics that have relevance to urban water-breeding mosquitoes (Elias et al., 2023). The survey involved 102 citizen science projects in Africa, and yielded 53 adequately completed responses (52% response rate). Results indicate that 56% and 44% of the projects reported involved SDGs 6 and 11, respectively. The survey also reveals that the primary purposes for the establishment of citizen science projects were to advance research (51%), to educate the public (43%), to ensure that informed policies are enacted (21%), and to capture data from end users (19%). Furthermore, the highest percentage of the 53 projects rely on in-kind institutional support from the host organization (19%) or do not have access to funding at all (19%), suggesting that much of the work is done on a voluntary basis. Funding sources for other projects include public-private partnerships (18%), donor organizations (13%), and governments (12%). The survey demonstrates that citizen science projects in Africa may suffer from insufficient or inconsistent sources of funds, hence compromising sustainability.

## RECOMMENDATIONS AND NEXT STEPS

### INVESTMENT

We therefore recommend that investment go toward building national and local infrastructure for citizen science networks focused on *An. stephensi* and malaria surveillance, including hiring community health workers, supplying materials (mobile devices, lenses, etc.), and supporting promotional campaigns (see below). Such crowd-sourced mosquito surveillance is a neglected but scalable and cost-effective solution, given its relatively low cost and non-recurring investments (Palmer et al. 2017). This recommendation is, of course, in addition to a call for greater investment in malaria control and elimination in Africa in general, as the gap between resource need and funding has dramatically widened over recent years to $3.8 billion in 2021 (WHO 2022b). Much of this support will need to come from international sources, from which 67% of total malaria funding was provided between 2010 and 2021 (WHO 2022b). Additionally, and with an eye toward building national capacities over the longer term, we echo the recommendation for some debt forgiveness for those countries investing in research and development (Nature 2023), particularly on issues of malaria, SDGs 6 and 11, and energy.

### ACCESS

Indeed, one fundamental challenge is that access to electricity is lacking for a staggering 43% of the continent’s population, approximately 600 million people (World Bank 2023). Another bottleneck includes the availability of devices necessary to download the 16–66 MB apps or to access GMOD and mosquitoID.org via the browser. This is especially true in low-resource and remote regions. In communities with low smartphone penetration, one recommendation is that a community health worker(s) be equipped with a smartphone or tablet. Through door-to-door or other campaigns, they could take photos of *Anopheles* mosquitoes on behalf of others in the community without a smartphone, and then submit the observations through the appropriate app (or upload photos from other individuals that have a camera).

Generally, however, smartphones are increasing in use, especially in urban areas where *An. stephensi* is expected to have the greatest introduction and impact. In Sub-Saharan Africa alone, there were over half a billion smartphone subscriptions in 2021, and this number is expected to reach 800 million by 2027 (statista.com 2022). Smartphones there represent approximately half of total internet connections, and mobile data consumption is expected to nearly quadruple by 2027 (Global System for Mobile Communications 2022).

Another critical bottleneck is the lack of access to magnification devices such as the 60× clip-on lenses for smartphones, which are required for imaging mosquito larvae (or diagnostic features for manual identification of *An. stephensi*, Figure 1). While to date our USF team has supplied more than 1,500 of these lenses to 18 African countries for the purpose of *An. stephensi* larval surveillance, these shipments take weeks to months, and some have been lost in transit. Alternative devices such as a high-powered hand magnifying glass, a microscope, or even reversed binoculars may serve to fill this need.

Community outreach and educational resources that can be used to expand the reach of citizen science mosquito monitoring programs are available from several sources, including Mosquito Habitat Mapper (https://observer.globe.gov/toolkit/mosquito-habitat-mapper-toolkit) and Mosquito Alert (https://www.mosquitoalert.com). It should also be noted that there are offline versions of many projects that could be deployed in areas with low or no broadband access. Several mobile apps for data reporting, such as Mosquito Habitat Mapper, do not need internet access to collect data; a signal is needed only to upload data to the digital archive. If citizen scientists are reluctant to use their personal credits for uploading such data, partnerships with local internet cafes or other establishments may provide a viable solution.

### MOBILIZATION

Similarly, data collection campaigns are most likely to be successful when strong partnerships are developed with local education, health, and touristic organizations, and where data collection can serve as a positive addition to existing programmatic goals. This is because even when the proper software, hardware, and educational resources are accessible, other bottlenecks and challenges can exist, such as training participants as well as sustaining their motivation to collect data. Community mobilization in urban areas is necessary to raise awareness of the problem and to foster the use of simple tools geared toward the identification of *An. stephensi*, such as 60× lenses coupled with our identification guide (Figure 1).

Concerted efforts should be made by stakeholders to recruit and mobilize app superusers and community health workers, and equip the latter with smartphones and lenses. With respect to community outreach and education surrounding such efforts, it would be prudent to leverage and scale existing networks such as school districts and scouting groups. It will also be strategic to repurpose existing arboviral surveillance programs—especially those targeting other container-breeding mosquitoes such as *Ae. aegypti* and *Ae. albopictus*, as was done for *An. stephensi* in Mauritius (Iyaloo et al. 2022). In particular, the Pan-African Mosquito Control Association (PAMCA; pamca.org) and the West African *Aedes* Surveillance Network (WAASuN; waasun.org) have *Aedes* surveillance programs that could be utilized, and perhaps coordinated and integrated with citizen scientist networks. Such integrated surveillance would align with the WHO Global Vector Control Response strategy (WHO 2017). Indeed, mosquito control agencies will be critical in mounting appropriate responses to *An. stephensi*, and their efforts could include monitoring and contributing to the GMOD. Additionally, gaps in the GMOD’s data in Africa reveal areas for targeting intervention from groups like CitSci Africa to promote as new places for citizen science uptake. These mobilization steps will help greatly in the surveillance stage, and if successful for a given area, can be scaled up rapidly to other parts of the continent.

### ENGAGEMENT

To greatly assist in scaling up mobilization efforts, promotional campaigns are also encouraged at the local, regional, and national levels. One such success story involves a national event on mainstream television and radio in the Netherlands on July 22, 2021, through which public figures urged citizens to use the Mosquito Alert app to help detect invasive *Aedes* species, and to a lesser extent, report bites. This led to an unprecedented surge in reports of mosquito adults and bites, which increased over 1,000-fold and 500-fold (5 versus 5.2 K adults, and 5 versus 2.7 K bites) in the week after the event compared with the week prior, with increased reporting observed through November 2021 (mosquitodashboard.org; reports without time/location data are omitted). If similar mosquito-targeted calls-to-action and mobilization could be achieved in African nations, it would certainly cast a much wider and denser net with which to catch *An. stephensi*—especially compared with traditional trapping methods. It would also have the benefit of increasing public education and awareness of this invasive vector and of malaria in general. Furthermore, such messaging would likely be efficient and cost-effective, given that media reach, smartphone penetration, and network effects of citizen scientists are all greatest in urban areas, exactly where this mosquito thrives.

Too often, projects created in the US and Europe fail to include voices from Africa, Central and South America, and Asia, an especially critical oversight when those are the countries where such tools are needed the most. Those blind spots in the initial project structure’s lack of representation can be ameliorated through deliberate inclusion and engagement with stakeholders and research partners from a truly international community, concerted collaboration with global mosquito tracking apps, and capacity-building efforts at the local grassroots level. Strengthening the partnership between groups like GMAC and CitSci Africa may be the most important first step to facilitating the transfer of such research knowledge (CitSci Africa 2021). Furthermore, for the citizen science infrastructure that already exists, such as the mobile apps and educational resources, it will be important to add African languages to better engage the necessary communities and to increase inclusivity. In particular, this involves translation into Swahili, which our group has recently completed.

### TESTING

It is important to communicate to citizen scientists not just the public health utility of the data being collected (Fischer et al. 2021), but also the necessity of testing any specimens that are suspected to be *An. stephensi* in a new area. Since 2020, the Mosquito Alert app has included instructions for mailing adult mosquito specimens to reference laboratories in selected countries, and this could be extended to any facility in Africa willing to participate. Through this process, users are notified through the app if they are in a country with laboratories actively accepting specimens (Figure 2). Such notifications could be sent only to those users submitting photos of *Anopheles* or unknown mosquitoes, and/or based on specific months or resource availability. It is worth noting that this strategy of having participants mail in mosquito specimens has been employed by at least a dozen other citizen science programs (Sousa et al. 2022). At present, full sequencing is the gold standard and the only acceptable method, as opposed to other methods such as polymerase chain reaction (Singh et al. 2023), for confirming *An. stephensi* in a new area (WHO 2023b). Until local testing capacity or other submission pipelines are developed, there will remain a critical bottleneck for the confirmation of *An. stephensi* in new areas and the deployment of necessary responses.

## CONCLUSIONS

*An. stephensi* is an accelerating threat in Africa, and there is an urgent need to detect the species in new areas (prior to establishment, for eradication) and to monitor existing populations (after establishment, for management). However, traditional mosquito surveillance such as trapping is expensive, requires expertise and time, and is not easily scalable, especially across jurisdictional boundaries. Citizen science should thus be a priority for both local communities and international health bodies like the WHO to complement ongoing *An. stephensi* surveillance measures in hard-to-reach and densely populated regions. Indeed, citizen science surveillance is a cost-effective, scalable, and sustainable solution that can leverage existing technological infrastructure (apps, GMOD) as well as local mosquito programs (especially those already targeting container-breeding mosquitoes). The ideal implementation would include both top-down and bottom-up approaches:

- **National and municipal media** would broadcast the message for citizens to download and use the app(s) to detect this invasive mosquito and fight the spread of malaria in their communities.
- **Mosquito control programs** and **public health agencies** would monitor GMOD (mosquitodashboard. org); follow up with surveillance and control when and where necessary (e.g., *Anopheles* larva in an artificial container); utilize our AI app (mosquitoID.org), and engage with citizen science networks and community health workers, perhaps equipping them with a smartphone and 60X clip-on lens.
- **Citizen science networks** and **community health workers** would mobilize and engage with their communities and especially superusers of the app platforms; coordinate training and bioblitz campaigns, leveraging existing online resources; and take and/or upload photos of *Anopheles* mosquitoes on behalf of others without a smartphone.
- **Citizen scientists** would contribute photos and information via our various apps, join our iNaturalist campaign (mosquitoesinAfrica.org), mail *Anopheles* specimens for confirmation sequencing, and recruit others in their community to participate.

Such comprehensive deployment would help achieve the goals of the WHO’s *An. stephensi* initiative and global vector response strategy (WHO 2017, 2022c). Ultimately, it is our hope that the free tools and resources that our coalition provides can serve to enable and embolden citizen scientists, as well as to inform scientists, mosquito control personnel, and policymakers in the accelerating fight against *An. stephensi* in Africa.
